# Fully spin-polarised quantum Hall effect at sub-tesla magnetic fields

**DOI:** 10.1038/s43246-026-01244-4

**Published:** 2026-07-03

**Authors:** M. Myronov, W. Jiang, S. Studenikin

**Affiliations:** 1 Physics Department, The University of Warwick, Coventry, UK; 2 National Research Council of Canada, Ottawa, ON Canada

**Keywords:** Quantum Hall, Electronic devices, Quantum metrology

## Abstract

The integer quantum Hall effect provides one of the most precise and robust manifestations of topological quantum transport. Yet, in semiconductor systems, its development to a fully spin-polarised state typically requires magnetic fields of several tesla and milli-Kelvin temperatures to overcome disorder, Landau-level broadening, and thermal smearing. Here we report a fully spin-polarised *ν* = 1 quantum Hall state in a two-dimensional (2D) hole gas hosted in a compressively strained germanium quantum well grown on silicon, stabilised at magnetic fields as low as 0.25 − 0.3 T and temperatures up to 1.5 K. This unusually low-field quantisation is enabled by an ultra-dilute carrier regime combined with extremely large Zeeman energy that approaches the cyclotron energy of free carriers over a wide density range, resulting in dominant spin splitting of Landau levels at sub-tesla fields to reach *ν*= 1 fully spin-polarized quantum limit. Magnetotransport and 2D Landau-fan measurements demonstrate that Landau-level broadening remains smaller than the spin splitting throughout the whole magnetic field range down to 0.25 mT, allowing robust quantisation of the Hall resistance at *h*/*e*^2^. These results establish access to an experimentally unexplored regime of spin-polarised quantum Hall physics in a semiconductor platform and open new opportunities for exploring exchange-driven and correlated many-body states of dilute systems in small magnetic fields.

## Abstract (web summary)

The integer quantum Hall effect is a precise manifestation of topological quantum transport, but achieving a fully spin-polarised state in semiconductors typically requires high magnetic fields and low temperatures. Here, the authors demonstrate a fully spin-polarised ν = 1 quantum Hall state in a germanium quantum well at low magnetic fields, enabled by ultra-dilute carriers and large Zeeman energy.

## Introduction

The quantum Hall effect (QHE) remains one of the most striking manifestations of quantum mechanics in condensed-matter systems, providing a direct connection between topology and electrical transport^1,2^. Since its discovery, it has served as both a paradigm for topological phases of matter and the foundation of a primary electrical-resistance standard^3–6^. In conventional semiconductor two-dimensional (2D) systems, however, the formation of fully developed quantum Hall states typically requires magnetic fields of several tesla and millikelvin temperatures, reflecting the need to overcome disorder, Landau-level broadening, and thermal fluctuations^5,7–9^.

Achieving robust quantisation at substantially lower magnetic fields has long been a central challenge. In particular, stabilising the fully spin-polarised *ν* = 1 state at low fields requires not only ultralow disorder, but also a hierarchy of energy scales in which the Zeeman splitting exceeds both the orbital energy and the disorder-induced Landau-level broadening^7,10–12^. While such conditions can be realised in a limited number of III–V electron systems^13,14^, they have remained inaccessible in group-IV semiconductor platforms on silicon. In conventional III–V semiconductor systems, strong and unavoidable Dresselhaus spin-orbit interaction intrinsically entangles spin and orbital degrees of freedom, complicating the isolation of purely spin-driven phenomena^15–17^. By contrast, centrosymmetric group-IV platforms suppress bulk spin-orbit coupling^18,19^, allowing large Zeeman splitting to dominate over orbital quantisation^11,12^. In this regime, a fully spin-polarised *ν* = 1 quantum Hall state emerges at magnetic fields below 0.3 T, enabling direct access to exchange-enhanced spin gaps and quantum Hall ferromagnetism in an exceptionally dilute, low-field limit.

Compressively strained germanium quantum well grown on silicon (cs-GoS) have recently emerged as an exceptionally clean host for two-dimensional hole gas (2DHG) with very small spin-orbit interactions, combining ultrahigh mobility with a small effective mass and a large effective g*-factor^11,12,20^. These properties suggest that, in the dilute regime, spin splitting may dominate orbital quantisation, opening a route to accessing spin-polarised quantum Hall physics at magnetic fields far below those required in conventional systems. Coupled with advances in epitaxial strain engineering and interface perfection, cs-GoS platforms now routinely exhibit hole mobilities exceeding 7 × 10^6^ cm^2^V^−1^s^−1^, surpassing many III–V systems, while maintaining CMOS compatibility and wafer-scale uniformity^20^. These attributes position cs-GoS as an exceptional environment for studying strongly correlated and topologically non-trivial states within a technologically mature materials framework.

Earlier studies of cs-Ge QWs have reported integer quantum Hall states at magnetic fields above 3–10 T, supported by well-resolved Shubnikov–de Haas oscillations^11,13,21–23^. However, achieving full spin polarisation and complete quantisation in the sub-tesla regime has remained unreachable, limited by the host material quality, interface disorder, background impurity fluctuations, and residual charge inhomogeneity that broaden spin-split Landau levels. Demonstrating full spin polarisation at sub-Tesla fields would signal not only a new benchmark in material purity, but also open an unexplored regime for probing quantum criticality, exchange-driven spin physics, and low-field topological phenomena.

Here we demonstrate a fully spin-polarised *ν* = 1 quantum Hall state in a cs-GoS 2DHG at magnetic fields as low as 0.25−0.3 T and temperatures up to 1.5 K. By tuning the carrier density into an ultra-dilute regime with mobile carrier densities below 10^10 ^cm^−2^ and exploiting the large Zeeman energy of holes in cs-GoS, we access a regime in which Landau-level broadening remains smaller than the spin splitting throughout the relevant field range, enabling robust quantisation at unprecedentedly low magnetic fields including very extended first fully spin-polarised QHE plateau *ν* = 1, a regime typically inaccessible in semiconductor systems^5,7,8,24,25^. The ultra-dilute regime denotes a low-density 2D system in which Coulomb and exchange interactions dominate over kinetic and orbital energies, enabling interaction-driven spin order at exceptionally small magnetic fields.

Very stable gated Hall-bar devices were fabricated employing the Schottky-gate technology from state-of-the-art cs-GoS epiwafers grown by reduced-pressure chemical vapour deposition and characterised via low-temperature magnetotransport measurements between 300 mK and 1.5 K. By tuning the top-gate voltage, we reliably accessed the extremely low hole density transport regime where Zeeman spin splitting dominates cyclotron energy ($\hslash {\omega }_{c}$) and level broadening when only odd-filling factor spin quantum oscillations remain. Under these conditions, the longitudinal resistance collapses to zero while the Hall resistance quantises to *h*/*e*^2^, demonstrating the complete formation of the fully spin-polarised *ν* = 1 quantum Hall state at magnetic fields down to 0.3 Tesla, easily achievable using simple permanent magnets. This behaviour is explained by a phenomenological model of Gaussian-broadened spin-resolved Landau levels with a large effective g*-factor. The results establish cs-GoS as a uniquely clean group-IV platform for exploring low-energy quantum Hall physics and spin-driven many-body states.

## Results

### Electrostatics and ultra-dilute regime

**Fig. 1:**
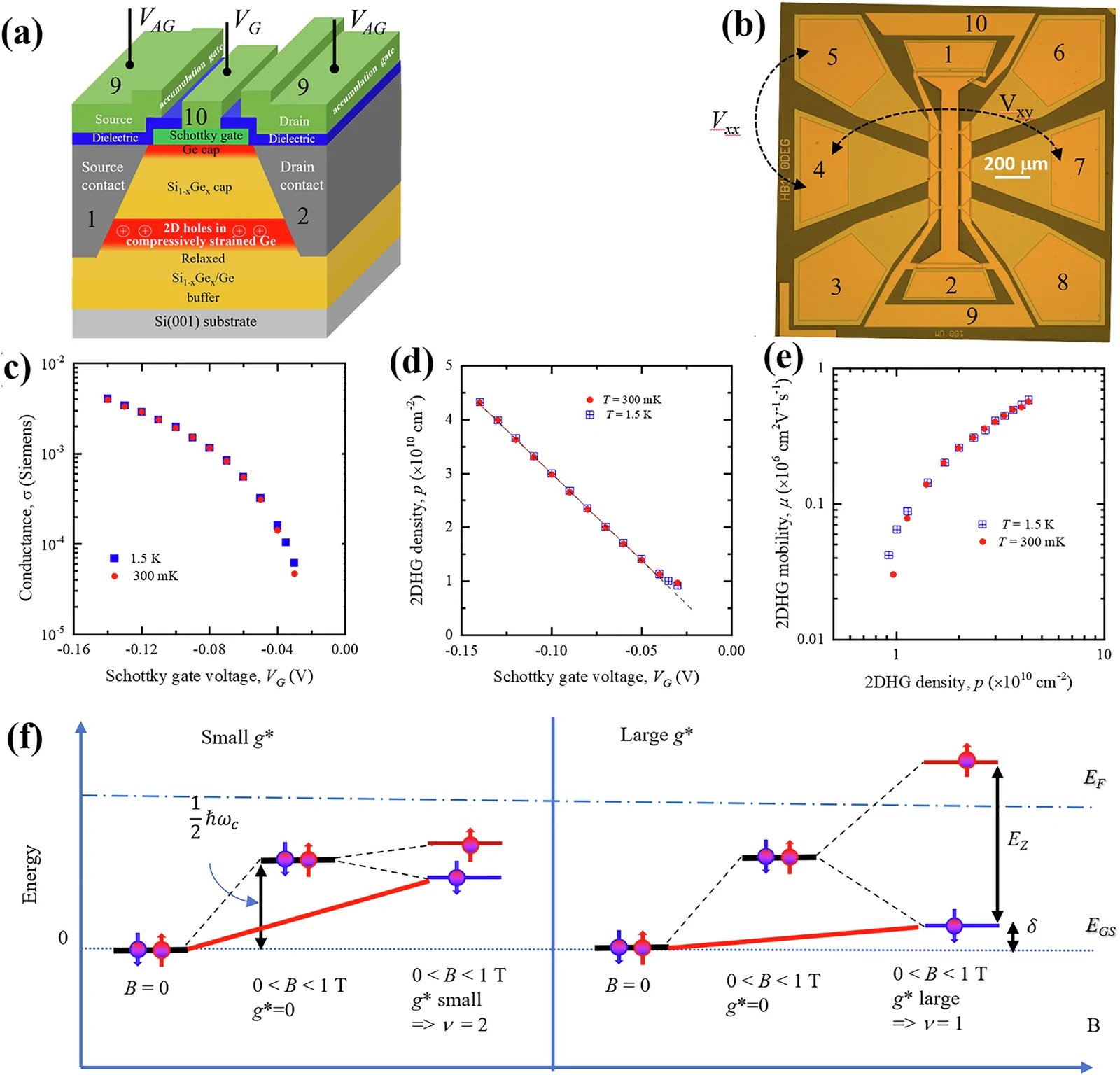
Device architecture, fabrication, and electrostatic characterisation of the ultra-low-density cs-GoS 2DHG. **a** Cross-sectional schematic of the cs-GoS heterostructure and Schottky-gated Hall-bar device. The 2DHG resides in a 30 nm wide cs-Ge QW, capped with 300 nm Si_1-x_Ge_x_ epilayer and contacted laterally through source and drain contacts. A thin Ge cap epilayer and an ALD-grown dielectric electrically isolate the central Schottky gate, made of Ti, while independent accumulation gates at the source and drain ensure low-resistance Ohmic contacts. The heterostructure is supported by a relaxed Si_1−x_Ge_x_/Ge buffer stack grown on a Si(001) substrate. **b** Optical micrograph of the fabricated gated Hall-bar device used for low-temperature magnetotransport measurements. The central mesa defines a long, narrow channel for high-precision Hall and longitudinal-resistance measurements, with eight Ohmic contacts arranged symmetrically around the active region. Scale bar is 200 μm. **c** Gate-voltage dependence of the device conductance at 1.5 K and 300 mK, demonstrating smooth and monotonic depletion of the 2DHG with no evidence of leakage or hysteresis. **d** Extracted 2D hole density *p* as a function of Schottky gate voltage, showing a linear and highly uniform electrostatic response at both temperatures. The low-density regime down to ∼1 × 10^10 ^cm⁻² is accessed with excellent stability. **e** Mobility *μ* versus carrier density *p*, revealing exceptionally high mobility exceeding 4 × 10^5 ^cm² V⁻¹ s⁻¹ at densities 9 × 10^9 ^cm⁻². The strong increase of *μ* with increasing *p* reflects the suppression of impurity and interface-roughness scattering in the ultra-clean cs-Ge QW. **f** Schematic energy diagrams of the lowest two spin levels of the 2DHG with respect to the ground state at *B* = 0 in different conditions, for a small effective g*-factor left part of the diagram and a large effective g*-factor, on the right. The right diagram explains why *ν* = 1 QHE plateau is very extended in our material system with a large g*-factor (for details see text).

Electrostatic drift and hysteresis, commonly observed in MOS-gated devices due to interface trap states, severely limit reproducible carrier-density control^20^. Here, we demonstrate stable and precise electrostatic tuning of the 2DHG using Schottky-gated Hall-bar devices fabricated from fully epitaxial cs-GoS heterostructures (Fig. 1). Continuous tuning of the carrier density down to *p* ≲ 1 × 10^10 ^cm^−2^ is achieved with negligible leakage and no measurable hysteresis, allowing systematic access to the ultra-dilute regime. Over this range, the density-gate relationship remains linear and stable across temperature range, indicating a highly uniform and stable electrostatic environment. By sweeping the Schottky top gate *V*_*G*_, we tune the hole density continuously from the dilute regime *p* ≈ 8 × 10^10 ^cm^−2^ into the extreme dilute transport regime *p* ≲ 1 × 10^10 ^cm^−2^. Over the full accessible range, the conductance decreases smoothly and monotonically determined at each point from Hall effect measurement (Fig. 1c), indicating very stable electrostatic gate control with negligible leakage and no measurable hysteresis or drift. The extracted density-gate relation remains strictly linear at both 300 mK and 1.5 K (Fig. 1d), revealing excellent uniformity of the electrostatic environment and stability over time and temperature.

As the 2DHG density is increased (Fig. 1e), the mobility increases sharply, reflecting the screening and therefore suppression of impurity and interface-related scattering and confirming the exceptionally low disorder of the system. This combination of ultra-low carrier density and minimal broadening establishes the conditions required for the formation of a fully spin-polarised system, i.e. $\nu \le 1,$ at magnetic fields far below those typical for semiconductor quantum Hall systems. At low densities, the mobility increases sharply, exceeding 42,000 cm^2^V^−1^s^−1^ at *p* ≈ 0.9 × 10^10 ^cm^−2^ (Fig. 1e). This behaviour reflects the very weak scattering centres present in the cs-Ge QW heterostructure, consistent with the absence of parallel channels, the high structural perfection of the cs-Ge epilayer interfaces, and the lack of remote-ionised impurity scattering within the heterostructure due to the undoped heterostructure design^20^. The exceptionally low disorder establishes the conditions under which Landau-level formation and spin resolution can occur at much lower magnetic energies in small magnetic fields than in any previously reported Germanium (Ge), Silicon-Germanium (Si_1−x_Ge_x_) or Silicon (Si) gated systems^11,21–23^.

Despite the extremely low carrier density, the system remains in a fully delocalised free-carriers transport regime. This is evidenced by the smooth evolution of conductance with gate voltage, the absence of mesoscopic fluctuations and weak localization effects, and the high carrier mobility retained down to *p* ≈ 0.9 × 10^10 ^cm^−2^ with the measured percolation density threshold *p*_*c*_ = 5 × 10^9^cm^−2^. The linear density–gate relation and the uniform Landau fan further indicate minimal spatial inhomogeneity and negligible charge trapping and puddling.

The lower panel of Fig.1f schematically illustrates the evolution of the lowest spin-resolved energy levels of the 2DHG relative to the zero-field ground state under different spin-splitting conditions. Zero energy is defined with respect to the doubly degenerate spin ground state (spin-up and spin-down) occupying the lowest subband of the quantum well at *B* = 0. Upon application of a magnetic field, the orbital ground state shifts upward due to the Landau diamagnetic shift at a rate of $\frac{1}{2}\hslash {\omega }_{c}$, where ℏ is reduced Plank constant and ${\omega }_{c}={eB}/{m}^{* }$ is the angular cyclotron frequency.

In the limit of a small effective g* factor (left panel), the Zeeman splitting ${E}_{Z}={{\rm{g}}}^{* }{\mu }_{B}B$ remains much smaller than the cyclotron energy $\hslash {\omega }_{c}$, resulting in weak spin resolution and correspondingly narrow *ν* = 1 quantum Hall plateau. By contrast, the right panel depicts the regime of a large effective g*-factor, where the Zeeman energy becomes comparable to the cyclotron energy (${E}_{Z}\approx \hslash {\omega }_{c}$). In this case, the spin-down branch is pushed downwards relative to the orbital ladder and evolves with the magnetic field at a reduced rate given by (see Methods):

$${\delta }=\frac{1}{2}{\hslash }{\omega }_{c}-{g}^{* }\,{\mu }_{B}\,B={\mu }_{B}\,({m}_{e}/{m}^{* }\,-{g}^{* }\,)B.$$

As a result, the lowest spin-resolved Landau level remains well below the Fermi level over a wide magnetic-field range, leading to a particularly flat and extended fully spin-polarised *ν* = 1 quantum Hall plateau, persisting even at relatively small magnetic fields and elevated temperatures. Experimental observation of this Zeeman-dominated regime is presented in the following section.

### Observation of the ν = 1 quantum Hall effect at sub-tesla magnetic fields

**Fig. 2:**
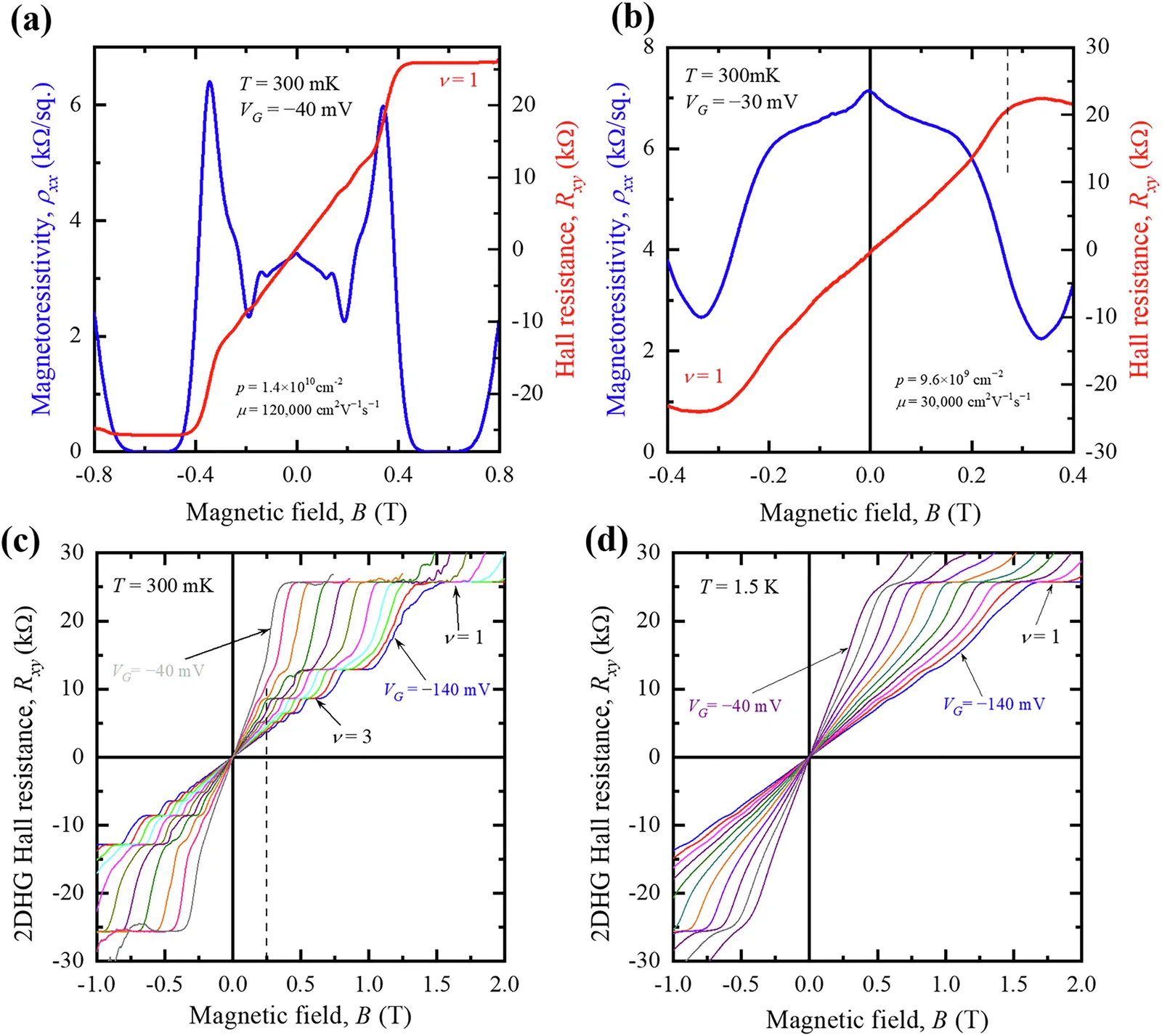
Emergence of the *ν* = 1 quantum Hall state at sub-tesla magnetic fields in an ultra-low-density cs-GoS 2DHG. **a** Magnetoresistivity *ρ*_*xx*_(blue) and Hall resistance *R*_*xy*_(red) measured at T = 300 mK and *V*_*G*_ = − 40 mV, corresponding to a 2D hole density *p* = 1.4 × 10^10^ cm^−2^ and mobility *μ* ≈ 120,000 cm^2^V^−1^s^−1^. A wide Hall plateau at *R*_*xy*_ = h/e^2^ is observed for ∣*B*∣ ≳ 0.4, accompanied by a deep minimum in *ρ*_*xx*_, signalling the formation of a fully resolved *ν* = 1 quantum Hall state at remarkably low magnetic fields. **b** Low-field magnetotransport at T = 300 mK and *V*_*G*_ = − 30 mV for an even lower density *p* = 9.6 × 10^9 ^cm^−2^ with mobility *μ* ≈ 30,000 cm^2^V^−1^s^−1^. The *ν* = 1 plateau remains clearly visible around *B* ≈ 0.25 T, demonstrating that quantisation persists deep into the ultra-dilute regime. The vertical dash line marks the field at which the plateau starts. **c** Hall resistance *R*_*xy*_ at T = 300 mK for the same sequence of gate voltages as in panel (**c**). At this temperature, both the *ν* = 1 and *ν* = 3 plateaux are resolved, with the *ν* = 1 state again emerging at ∣*B*∣∼0.25–0.3 T. The sharpening and widening of the plateaux relative to the 1.5 K data reflect reduced thermal broadening and highlight the exceptional material uniformity and Landau-level isolation in the cs-GoS system. **d** Hall resistance *R*_*xy*_ measured at *T* = 1.5 K for a series of top-gate voltages *V*_*G*_ between –0.14 V and –0.04 V, corresponding to carrier densities spanning the range *p*∼(0.8–4.0) × 10^10 ^cm^−2^. As *V*_*G*_ is tuned towards less negative values (lower density), the *ν* = 1 plateau broadens systematically and shifts to lower magnetic fields, consistent with an increasing ratio of Zeeman to cyclotron energy.

Figure 2a shows an example of the longitudinal magnetoresistivity, *ρ*_*xx*_, and Hall resistance, *R*_*xy*_, measured at *T* = 300 mK for a density *p* = 1.4 × 10^10 ^cm^−2^. Even in this ultra-dilute regime, the system exhibits clear signatures of Landau quantisation at surprisingly low magnetic fields. As the field increases to ∣*B*∣ ≈ 0.4 T, *ρ*_*xx*_ collapses to zero while *R*_*xy*_ develops an unusually broad plateau at h/e^2^, demonstrating the complete formation of the fully spin-polarised *ν* = 1 quantum Hall state at low magnetic field. This critical field is more than an order of magnitude lower than values reported for earlier SiGe-based 2D hole gases, and lower even than typical GaAs/AlGaAs high-mobility electron or hole systems, which normally require several tesla to achieve a fully polarised *ν* = 1 spin state and milli-Kelvin temperatures^26^.

At a slightly lower density *p* = 9.6 × 10^9 ^cm^−2^, the *ν* = 1 plateau still persists and shifts to even smaller magnetic fields (Fig. 2b). The appearance of a robust quantised Hall plateau at ∣*B*∣ ≈ 0.25–0.30 T confirms that the Zeeman energy is comparable with the cyclotron energy, placing the system in a regime where the ground state is pushed down. That makes the fully spin-polarised *n* = 1 QHE plateau very extended even in small fields below 1 T and observable at relatively large temperatures above

1.5 K in our experiments. By contrast, in undoped GaAs two-dimensional hole systems, the *ν* = 1 state typically collapses below magnetic fields of ~2 T at comparable temperatures^26^.

The extended *ν* = 1 plateau observed here is a particularly significant feature. First, its stability at magnetic fields well below 1 T, down to approximately 0.3 T, enables access to a fully developed quantum Hall state using modest magnetic fields, including those achievable with permanent magnets. More importantly, from a fundamental perspective, this low-field regime realises a fully spin-polarised two-dimensional hole system in which all carriers occupy the same spin ground state. Such a configuration provides a clean and well-defined starting point for exploring collective spin phenomena and spin-resolved many-body physics in the absence of competing spin-orbital effects. Finally, the hierarchy of energy scales achieved here, in which the Zeeman splitting exceeds the Landau-level spacing, creates favourable conditions for investigating exchange-driven and ferromagnetic ordering phenomena in an exceptionally low-disorder environment. This regime of a fully spin-polarised collective state at sub-tesla magnetic fields has not, to our knowledge, been previously observed in semiconductor quantum wells based on CMOS-compatible materials.

### Gate-tuned evolution of spin-resolved Landau levels

To map the evolution of the quantum Hall states with density, we acquire gate-dependent Hall traces at fixed temperatures of 300 mK and 1.5 K (Fig. 2c, d). As the gate voltage is made less negative, reducing the density towards the minimal values, the *ν* = 1 plateau strongly widens while its onset shifts to lower magnetic fields. This behaviour is consistent with the qualitative model depicted in Fig.1f, right diagram for large effective g^*^-factor, when Zeeman energy dominates over the orbital quantization^11^.

At 300 mK, the plateau edges sharpen further, and higher-order odd plateaux (*ν* = 3) become visible extending down to *B* = 0.25 T for the corresponding gate voltages (Fig. 2c). The robustness of the *ν* = 1 state at sub-tesla magnetic fields and finite temperatures up to at least 1.5 K demonstrates that disorder-induced broadening of LLs remains extremely small, of order 40 μeV, across the full density range studied, confirmed by the modelling below.

#### Landau-fan analysis and energy hierarchyA

**Fig. 3:**
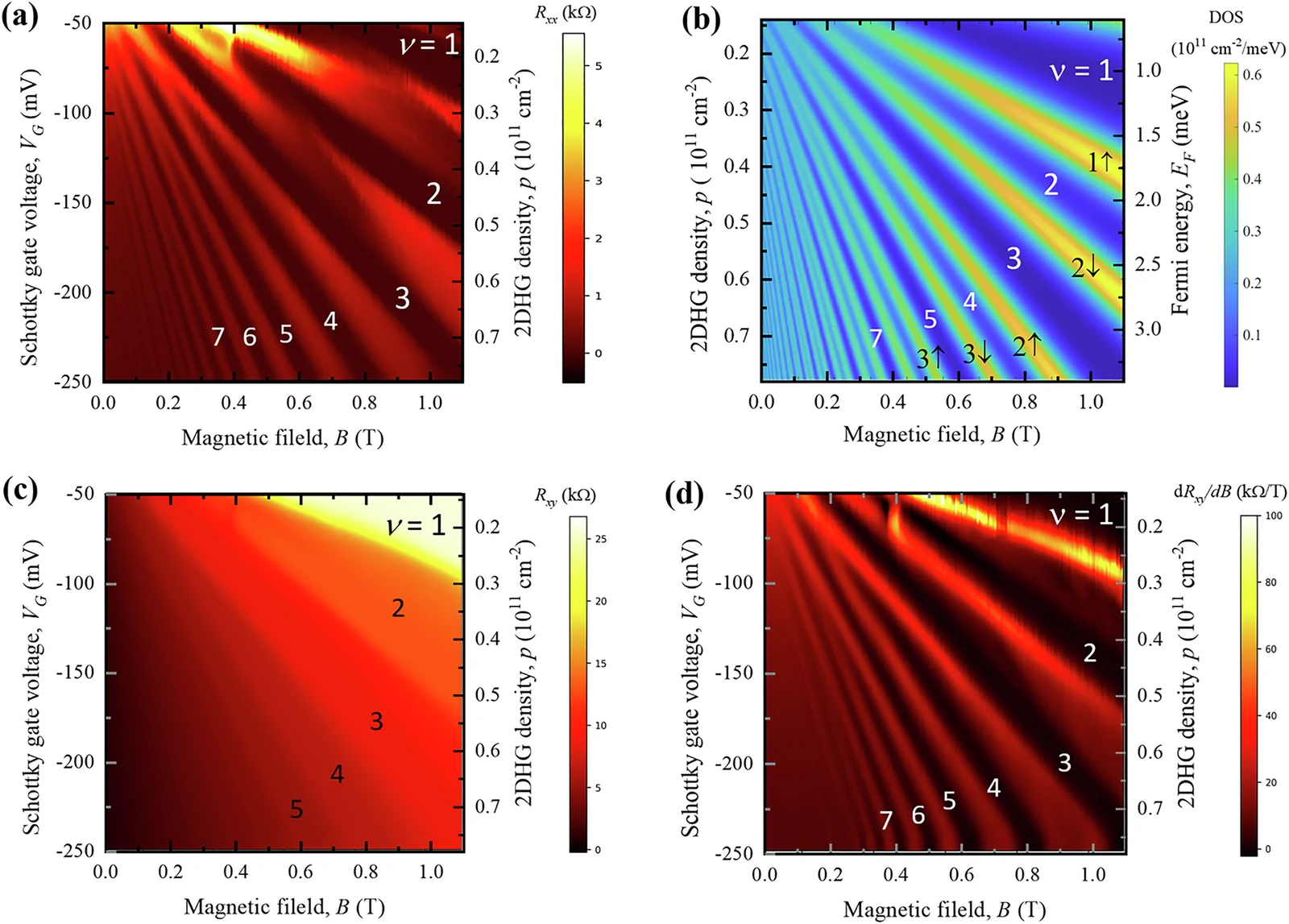
Landau-level fan diagram and spin-resolved quantum oscillations in the ultra-low-density cs-GoS 2DHG. **a** Colour map of the magnetotransport longitudinal resistance, *R*_*xx*_ as a function of magnetic field *B* and the Schottky gate voltage, *V*_*G*_, measured at *T* ≈ 300 mK, the right axis is equivalent 2DHG density from Fig. 1d defined only by the material stack and not affected by any surface charges. Multiple bright diagonal features form a well-resolved Landau-level fan emanating from the dilute limit (2DHG *p*_*G*_ ≈ 0.14–0.78 10^11 ^cm^−2^). The strong linearity and uniform spacing of the trajectories indicate exceptionally low disorder and high microscopic, spatially homogeneous carrier density distribution across the large area of the device. The pronounced splitting of adjacent lines for odd filling factors, *ν* = 3, 5, 7.., reflects the dominance of Zeeman energy over the orbital splitting. **b** Simulation of the Gaussian shape spin-split Landau levels with parameters close to the experimental situation, g*=15, m*=0.055, and quantum relaxation time *τ*_*q*_ = 8.2 ps or equivalent quantum mobility ~2.6 × 10^5^ cm^2^V^−1^s^−1^, or LL broadening parameter $\Gamma =40\,\mu {eV}$ (For details see Methods). **c** Hall effect resistance measured simultaneously with *R*_*xx*_. Clear integer QHE plateaus are evident in agreement with line traces in Fig. 2. **d** Differential Hall resistance d*R*_*xy*_/d*B* showing the same set of dispersing spin-split Landau levels with enhanced contrast. The sharp intensity maxima trace spin-resolved Landau branches, whose separation remains nearly constant over the full magnetic-field range up to *B* ≈ 1 T. The clarity of the features demonstrates that Landau-level broadening remains smaller than the Zeeman splitting throughout this regime, consistent with the emergence of the *ν* = 1 quantum Hall plateau at sub-tesla fields.

defining feature of the present system is the hierarchy of energy scales governing spin-split Landau quantisation. The Zeeman energy *E*_*Z*_ = *g*^*∗*^*μ*_*B*_*B*, cyclotron energy ℏ*ω*_*c*_ = ℏe*B*/*m*^*∗*^, and Landau-level broadening Γ satisfy *E*_*Z*_ ≈ ℏ*ω*_*c*_*/2* ≫ Γ over the studied density range. To qualitatively simulate or data we use *g*^*∗*^≈15 and *m*^*∗*^≈0.055m_0._ For small densities this condition allows us to reach the fully spin-polarised *ν* = 1 state in low magnetic fields down to B∼0.25–0.3 T, as evident in Fig.2c. The estimated in Fig.3b small Landau-level broadening Γ = 40 μeV ensures that spin splitting remains well resolved, enabling robust quantisation in a Zeeman-dominated regime in very small magnetic fileds.

To directly visualise the evolution of the spin-resolved Landau-level spectrum, we construct Landau-fan diagrams, shown in Fig. 3, by measuring the magnetotransport parameters *R*_*xx*_ and *R*_*xy*_ as a function of magnetic field and gate voltage. The resulting fan diagram exhibits well-resolved, linearly dispersing trajectories with dominant splitting between spin-resolved branches. The clarity and uniformity of these features indicate good uniformity and low disorder of the system.

Importantly, the separation between spin-resolved Landau levels remains larger than the orbital gap, *E*_*orb*_ = *E*_*c*_*-E*_*z*_, across the full density range explored. This hierarchy, in which the Zeeman energy dominates orbital quantisation, ensures that Landau-level broadening remains smaller than the spin splitting, providing a direct microscopic explanation for the robustness of the *ν* = 1 quantum Hall plateau at ultra-low magnetic fields.

Crucially, the pronounced splitting of adjacent branches reveals the separation between spin-up and spin-down Landau levels, confirming that the Zeeman gap exceeds the cyclotron gap throughout the low-density regime. This hierarchy stabilises a fully spin-polarised *ν* = 1 ground state at magnetic fields reachable not only by standard laboratory magnets but even by permanent magnets.

The differential Hall signal (Fig. 3b) enhances these features, resolving spin-resolved branches with remarkable sharpness. The separation between the two branches remains nearly constant with gate voltage, demonstrating that the large effective g*-factor at low density compensates the reduction in cyclotron energy, maintaining the Zeeman-dominated ordering across the entire density range tuned by gate voltage.

The Landau-fan structure reveals an unusual regime in which the Zeeman energy is comparable to, and in parts exceeds, the cyclotron energy, *E*_*Z*_ ≈ ℏω_c_. As a result, the lowest spin-resolved Landau level disperses only weakly with magnetic field, leading to an anomalously slow evolution of the *ν* = 1 ground state. This hierarchy strongly suppresses orbital depopulation while maintaining a large spin gap, providing a natural explanation for the exceptional width and robustness of the *ν* = 1 quantum Hall plateau observed at sub-tesla fields. Such behaviour is fundamentally distinct from conventional Si or GaAs-based systems with small effective g*-factors, and enables quantum Hall operation in magnetic fields accessible even with permanent magnets.

The effective g^*^-factor used in the modelling (g^*^≈15) is consistent with independent measurements in a similar cs-Ge QW platform, where a large and electric-field-tuneable g^*^-factor observed demonstrating a crossing between Zeeman and the orbital quantization energies (see Fig. 4b in ref. ^11^).

It should be noted, that in gated structures the metallic gate can essentially screen the electron-electron interaction, in particular, in ultra-dilute regime when distance between particles is comparable with the distance to the metallic gate^12,27^. This topic of free carrier exchange interactions requires a separate careful investigation which is beyond the scope of this work. In our experiments we do not observe strong evidences of additional g*-factor enhanced behaviour. Therefore, the experimentally observed values of g*-factor we interpret as an effective quantity, incorporating both single-particle and possible many-body contributions. The presented phenomenological model does not explicitly include density-dependent exchange enhancement, but captures the experimentally observed hierarchy of energy scales and overall behaviour. The good agreement between experiment and simulation indicates that a constant effective *g∗* provides an adequate first-order description of the observed quantization regime without additional fitting parameters.

### Implications for Zeeman-dominant quantum Hall physics at low magnetic fields

The combination of ultra-low disorder, dilute carrier density, and a very large effective hole g*-factor places the cs-GoS 2DHG in a new regime of quantum transport that has been remained largely unexplored experimentally. The observation of a fully spin-polarised *ν* = 1 quantum Hall state at magnetic fields as low as 0.25−0.3 T demonstrates access to a Zeeman-dominated quantum Hall regime in which spin splitting can exceed the orbital splitting energy between neighbouring Landau-level spin states. This hierarchy of energy scales enables stabilisation of a spin-polarised ground state under conditions where orbital quantisation alone would not be sufficient.

Access to this regime provides new opportunities to investigate many-body spin physics in the lowest Landau-level limit, including exchange-driven spin polarisation and quantum Hall ferromagnetism, under magnetic fields far below those typically required in semiconductor systems. More broadly, the ability to tune continuously between cyclotron- and Zeeman-dominated regimes in an exceptionally clean hole system offers a controlled platform for exploring spin-resolved many-body phenomena and low-energy quantum critical behaviour in two dimensions.

Finally, while the present work is primarily focused on fundamental physics, the realisation of a fully spin-polarised quantum Hall state at sub-tesla magnetic fields highlights the feasibility of accessing quantum Hall phenomena in compact cryogenic environments. This capability may enable future experimental studies of quantum Hall physics under reduced magnetic-field constraints, without altering the underlying physical principles that govern many-body phenomena and topological transport.

## Discussion

The observation of a fully spin-polarised *ν* = 1 quantum Hall state at magnetic fields below 1 T down to 0.25 T establishes access to a distinct regime of quantum Hall physics in a semiconductor system. In contrast to conventional two-dimensional platforms, where large magnetic fields are normally required to achieve spin-resolved states, the present system realises a qualitatively different hierarchy of energy scales in which the Zeeman splitting dominates over orbital quantisation. This regime emerges from the combination of ultra-low disorder, dilute carrier density, and the large effective *g*^∗^-factor of holes in cs-GoS material system.

In this Zeeman-dominated regime, the lowest spin branch of the lowest Landau level remains inactive, i.e., energetically isolated over a wide density range, even at sub-tesla magnetic fields. As a result, spin degeneracy is lifted before orbital quantisation, leading to robust formation of a fully spin-polarised ground state under conditions where conventional cyclotron-driven quantisation would be insufficient. This behaviour highlights a qualitatively different pathway to the formation of odd QHE states, governed primarily by spin physics.

Access to this regime enables systematic investigation of interaction-enhanced spin phenomena in the low Landau-level limit, including exchange-driven spin polarisation and quantum Hall ferromagnetism, under magnetic fields and cyclotron energies that are exceptionally small. The ability to tune continuously between cyclotron- and Zeeman-dominated regimes by carrier density/gate voltage provides a powerful experimental handle on the interplay between orbital quantisation, exchange interactions, and subband structure in hole systems with strong spin-orbit coupling. Owing to the stringent requirements on disorder and electrostatic stability, this limit has received comparatively little experimental attention to date.

More broadly, these results challenge prevailing assumptions regarding the magnetic-field scales required to realise topological quantum transport in semiconductor systems. They demonstrate that, with sufficiently low disorder and favourable spin properties, the spin-driven quantum Hall effect can persist deep into a low-energy regime where both orbital and spin quantisation occur at magnetic fields accessible to compact cryogenic environments. This expansion of the accessible phase space opens new opportunities to explore spin-resolved and correlated quantum Hall phenomena under conditions that have previously been difficult to achieve experimentally.

Taken together, our findings establish cs-GoS heterostructures as an exceptionally clean and tuneable platform for investigating low-field, spin-polarised quantum Hall physics. The combination of ultra-low disorder, large Zeeman splitting, and precise electrostatic control enables access to a Zeeman-dominated regime that provides new insight into the fundamental interplay between spin, topology, and interactions in two-dimensional hole systems.

We note that conventional Arrhenius activation-gap analysis is not directly applicable in the present low-field regime. The *ν* = 1 state appears at magnetic fields where thermal broadening and disorder-induced level broadening are comparable to the intrinsic gap, leading to transport dominated by percolation transport rather than a well-defined activation energy. Instead, the relevant energy scales are estimated from the Landau fan and the level broadening analysis (see Methods).

## Conclusions

In summary, we have demonstrated a fully spin-polarised *ν* = 1 quantum Hall state in the cs-GoS 2DHG at magnetic fields as low as 0.25−0.3 T and temperatures up to 1.5 K. This unprecedentedly low-field quantisation arises from an unusual hierarchy of energy scales in the ultra-dilute regime, where the Zeeman splitting exceeds both the cyclotron energy and Landau-level broadening. These results establish access to a new regime of spin-polarised quantum Hall physics in a semiconductor platform and open pathways for exploring correlated many-body states at ultra-low magnetic fields. The unusually low-field fully spin-polarised regime arises from the combined effect of very large Zeeman splitting and ultra-low disorder allowing to reach very small carrier densities. The two conditions are essential to reach very small values for the magnetic-filed quantum limit reaching the ν = 1 state in very small fields down to 0.25 T.

These findings establish cs-GoS as one of the cleanest and most spin-responsive group-IV quantum-well platforms reported to date. The ability to stabilise and tune quantum Hall quantisation using magnetic fields attainable with a permanent magnet opens compelling opportunities for compact metrological devices, cryogenic CMOS integration, and the exploration of interaction-driven phenomena such as quantum Hall ferromagnetism, Wigner crystallization, and spin-texture excitations. More broadly, our results demonstrate that advanced epitaxial engineering within the silicon materials ecosystem can yield platforms that rival and, in key regimes, surpass the performance of established III–V quantum Hall systems. This work also establishes the material foundation for further investigations of low-energy topological states in scalable, CMOS-compatible architectures.

## Methods

### Heterostructure growth and materials characterisation

The cs-GoS heterostructures were grown on 150 mm diameter Si(001) wafers using reduced-pressure chemical vapour deposition (RP-CVD). A schematic cross-section of the heterostructure and the fabricated Schottky-gated Hall bar device, showing the source (S), drain (D) and the gate (G) stack, is presented in Fig. 1a. The thickness of the Si(001) wafer is standard, i.e. ~700 µm. A graded Si_1-x_Ge_x_/Ge buffer of ~4 µm thick was first deposited to induce close to full relaxation of the Si_1-x_Ge_x_/Ge/Si(001) virtual substrate while maintaining threading-dislocation densities below 5 × 10^6 ^cm^−2^. The active region consists of a~30 nm thick cs- Ge QW epilayer embedded between relaxed Si_1-x_Ge_x_ barriers, relaxed epilayers, with Ge content x = 0.85, capped with a~300 nm Si_1-x_Ge_x_ epilayer and a ~3 nm Ge surface cap epilayer. Root mean square surface roughness of the cs-GoS heterostructure is below ~2 nm. No intentional doping was introduced. Strain and composition were determined from analysis by high-resolution X-ray diffraction and XTEM of the grown materials. Surface morphology was verified by AFM. Under the application of a negative gate bias, holes accumulate and are confined within the 30 nm-thick undoped, cs-Ge QW, which acts as the active p-channel for mobile holes.

### Device fabrication

Double-gated Hall-bar devices were fabricated using standard UV photolithography, wet and dry etching, and thin-film deposition techniques. The fabrication sequence comprised scribe-channel definition, mesa etching, ohmic contact formation, Schottky gate deposition, accumulation-gate dielectric deposition, and top-gate metallisation.

At the start of the process, 50-µm-wide scribe channels were patterned and etched into the Si substrate. The scribe channels enabled precise and smooth separation of individual chips by dicing or cleaving, significantly improving die quality and yield.

The mesa was defined by photolithography followed by inductively coupled plasma (ICP) etching using a Cl_2_/Ar gas mixture. The total etch depth was 390 nm, extending beyond the cs-Ge QW. To achieve a sloped mesa profile that ensures continuous metal coverage along the sidewall, the chamber pressure, gas ratio, and RF power were optimised. In addition, a photoresist reflow step was applied by baking the Shipley photoresist at 190 °C for 1 min, yielding a mesa sidewall angle of ~68°.

Source, drain, and voltage probe contacts were subsequently defined by photolithography. Following a 30 s dip in 1% diluted HF to remove the native oxide, 120 nm of Al was deposited by electron-beam evaporation and patterned by lift-off. To obtain low-resistance ohmic contacts, the samples were annealed at 275 °C for 30 min in N_2_. Post-anneal structural analysis using scanning transmission electron microscopy (STEM) combined with energy-dispersive X-ray spectroscopy (EDX) revealed Al diffusion of ~20 nm into the Ge quantum well from the mesa sidewall. The resulting Al–Si_1−x_Ge_x_ alloy provides lateral electrical access to the 2DHG.

Schottky gate was then patterned on top of the mesa. After a 30 s dip in 1% diluted HF, a 20/100 nm Ti/Au metal stack was deposited by electron-beam evaporation, forming Schottky contacts that electrostatically modulate the carrier density in the compressively strained Ge quantum well. The Schottky-gated cs-GoS architecture avoids charge trapping associated with dielectric-semiconductor interfaces and enables precise carrier-density control without detectable hysteresis or threshold-voltage shifts. The gate width was 90 µm, and the gate edge was positioned ~5 µm away from the mesa edge, ensuring that the active channel region is defined electrostatically rather than by etching. This geometry minimises potential fluctuations at mesa edges and suppresses edge-related scattering effects^28^.

A 50-nm-thick Al_2_O_3_ dielectric layer was subsequently deposited by atomic layer deposition (ALD) at 200 °C, using trimethylaluminium (TMA) and water as the aluminium and oxygen precursors, respectively. This dielectric serves both as the accumulation-gate dielectric for ohmic contacts and as an electrical isolation layer between metal contacts and the substrate. Contact vias were opened by photolithography, followed by selective removal of the Al_2_O_3_ using dilute buffered oxide etch.

Finally, a 20/200 nm Ti/Au metal stack was deposited and patterned to form independent injection gates used to initialise the ohmic contacts. The Al_2_O_3_ layer electrically separates these gates from the cs-GoS heterostructure, while the Au capping layer provides a robust surface for wire bonding. Individual dies were separated by dicing along the scribe channels and mounted onto chip carriers for electrical characterisation.

### Low-temperature electrical measurements

In this study we explore longitudinal magneto-resistance, *R*_*xx*_, and the quantum Hall-effect resistance, *R*_*xy*_, acquired from the four-probe voltage measurements between corresponding potential probes across or along the Hall bar in Fig. 1b. The differential potentials were measured using two lock-in amplifiers to acquire *V*_*xx*_ and *V*_*xy*_ signals simultaneously. The excitation current through the source-drain *I*_*ac*_=1na was supplied by the reference signal of the master lock-in.

As evident from Fig.1a, b, and from the Device Fabrication, we use two independent gates to activate contacts, and control the active region of the Hall bar independently. This allowed us to reach very low 2DHG carrier densities, almost down to the percolation threshold, *p*_*c*_ = 5 × 10^9^cm^−2^. Note, that we measure Hall effect and conductance of the 2DHG free carrier at this low density that indicates good uniform quality of the material platform with not-screen electrostatic potential fluctuations less than 0.4 meV. The small level of electrostatic fluctuations is an important feature for quantum technologies, e.g. to avoid spontaneous formation of quantum dots^29^.

Sample was cooled down in a helum-3 Oxford Instruments 3He cryostat (HelioxAST VL) equipped with superconducting magnet with the magnetic field B directed perpendicular to the plane of the 2DHG structure.

### Data analysis and simulations

In our experiments we measure voltage drops along and across the Hall bar structure, *V*_*xx*_ and *V*_*xy*_ correspondingly as indicated in Fig.1b. From these measurements we extract all necessary parameters used in this study: (1) conductance at zero magnetic field $\sigma ={L}{I}_{ac}/({V}_{\!\!xx}w)\,$ where *L* is distance between potential probes, *w* is width of the Hall bar (Fig.1b), *V*_*xx*_ is the voltage drop between potential contacts indicated in Fig. 1b; (2) 2DHG density $p=B/e{R}_{{xy}}$ where ${R}_{{xy}}={V}_{{xy}}/{I}_{{ac}}$ is Hall resistance, *e* is elementary electron charge, *B* is the value of magnetic field at which this measurement was taken, in this study at 50 mT; (3) the 2DHG density is calculated from *R*_*xy*_, *p* = *B*/(*eR*_*xy*_); and (4) the 2DHG mobility $\mu =\sigma {R}_{{xy}}(B)/B$. These 2DHG parameters are used to create line plots shown in Figs. 1 and 2. In Fig. 3a we have created a full 2D map of *R*_xx_ (*B*) stepping Schottky gate voltage, which is uniquely linked to the 2DHG density (right axis) as shown in Fig.1d, because it is not affected by interface charges and was exactly reproduced in several cool-downs. This is a very desirable property for building large scale reproducible quantum devices. In Fig. 3c we plot a similar 2D map for *R*_*xy*_*(B)*, the main subject of this research, where odd and even QHE plateau are clearly seen and marked as *ν* = 1, 2,3,4,5. Phenomenologically, according to known empirical observation by Cang and Tsui^30^, the derivative of the Quantum Hall resistance, *R*_*xy*_, with respect to the carrier density or magnetic field, *dR*_*xy*_*/dB*, exhibits a strong similarity to the diagonal resistivity, *R*_*xx*_^31,32^. with the following relationship: $\beta {\rho }_{{xx}}={Bd}{R}_{{xy}}/{dB}$, where ${\rho }_{{xx}}=w{R}_{{xx}}/L$ is magnetoresistivity, and β is a constant that varies from sample to sample between 20 and 40. Based on this phenomenological relationship, for qualitative comparison, in Fig. 3d we plot differential *dRxy/dB* of the data plotted in Fig.3c. Comparing Figs. 3a and 3b, it is evident that they are qualitatively very similar with fan-line structures and the QHE valleys, marked as *n* = 1,2,3,4,5,6,7. There are subtle differences many-body exchange interactions^33–35^, discussion of which is beyond of the scope of this article. For qualitative explanation of the observed fan-diagram in Fig. 3a, we made its modelling based on simplified Gaussian-shape landau levels, width of which is determined by the quantum scattering time, ${\tau }_{q}$, or equivalently by the quantum mobility, ${\mu }_{q}=e{\tau }_{q}/{m}^{* }$, LL width (broadening) $\Gamma =\hslash /2{\tau }_{q}$ The Landau levels (LL) energy fan is described by the following equation: ${E}_{{LL}}\left(N,\sigma \right)=\sum \left(N+\frac{1}{2}\right)\hslash {\omega }_{c}\pm \sigma {E}_{Z}\,,$ where $\hslash {\omega }_{c}$ is the cyclotron energy with ℏ been the reduced Plank constant, ${\omega }_{c}=e{B/m}^{* }$, ${E}_{Z}={g}^{* }{\mu }_{B}B$ being Zeeman spin splitting energy, ${\mu }_{B}$ been the Bohr magneton, *N* = 0,1,2… been the Landau quantum number, and $\sigma =\pm \frac{1}{2}$ the spin quantum number. In our simulations to describe spin-resolved Landau levels we use Gaussian Density of States (DOS) defined as following^36–38^:

$${\rm{DOS}}(E,B)=({\rm{e}}/{\rm{h}})({2}{{\pi}} )^{-1/2}\left({\rm{B}}/{{\varGamma }}_{\rm{G}}\right)\sum {\rm{exp}} \left[\mbox{-}\left(E-E_{{LL}}\left(N, \sigma ,B\right)\right)^{2} /2{\Gamma }_{G}^{2}\right]$$

Where ${\Gamma }_{G}=\sqrt{{\hslash }^{2}{\omega }_{c}/2\pi {\tau }_{q}}$ being the LL Gaussian linewidth, ${\tau }_{q}$ – free particle quantum lifetime directly linked to quantum mobility ${\mu }_{q}=e{\tau }_{q}/{m}^{* }$, ${E}_{{LL}}(N,\sigma ,B)$ is the spin-resolved LL spectrum defined above. The simulated spectrum of Gaussian-broaden LLs is plotted in Fig.3b using parameters to have the image closely resemble the experimental fan-diagram on Fig.3a. We use ${m}^{* }=0.055{m}_{e}$ and ${g}^{* }=15$ determined in our earlier paper^12^, with *m*_*e*_ being the free-electron mass. The only adjustable parameter is quantum relaxation time ${\tau }_{q}=$ 8.2 ps or equivalent quantum mobility ${\mu }_{q}$ = 2.6−10^5^ cm^2^V^−1^s^−1^. The LLs in Fig. 3b are marked with their corresponding quantum numbers ${|N}{\sigma }{{\rangle}}$, the QHE odd and even valleys are marked with corresponding fully occupied sates *ν* = 1,2,3…

It should be emphasised that here we have an unusual situation when Zeeman split energy is large and close to the cyclotron energy, e.g., ${E}_{Z}{\approx }{\hslash }{\omega }_{c}$. In this situation the lowest ground state of the system ${|N}=0,\sigma =-\frac{1}{2}{{\rangle }}$ shifts very slow with the magnetic field that is illustrated in diagram Fig. 1f with the rate determined by the following equation:

$$\delta =\frac{1}{2}{\hslash }{\omega }_{c}-{{\rm{g}}}^{* }{\mu }_{B}B={\mu }_{B}\,\left(\frac{{m}_{e}}{{m}^{* }}-{{\rm{g}}}^{* }\right)B$$

The above equation can be rewritten in the following form have $\frac{\delta }{\hslash {\omega }_{c}}=\frac{1}{2}(1-\frac{{{\rm{m}}}^{* }{{\rm{g}}}^{* }}{{{\rm{m}}}_{{\rm{e}}}})$

For the given m^*^ and g^*^ parameters we have $\frac{\delta }{\hslash {\omega }_{c}}\approx 0.088,\,$ which is almost one magnitude slower compared to the grown-state Landau diamagnetic shift of spin unresolved and small effective g^*^-factor systems. This fact qualitatively explains why the *ν* = 1 plateau is so extended in plots of Fig. 2 compared to ordinary semiconductor systems like Si and GaAs/AlGaAs with small effective g^*^-factor. This feature is very useful for QHE CMOS-compatible resistance etalons in field applications^39^ using permanent magnets below 1 T and accessible temperatures in helium transport Dewar containers.

## Data Availability

All data supporting the findings of this study are available within the article. The raw data underlying the figures presented in this article are available from the corresponding authors upon request.
